# Perioperative outcomes in geriatric patients undergoing hip fracture surgery with different anesthesia techniques: A systematic review and meta-analysis

**DOI:** 10.1097/MD.0000000000018220

**Published:** 2019-12-10

**Authors:** Dong Xu Chen, Lei Yang, Lin Ding, Shi Yue Li, Ya Na Qi, Qian Li

**Affiliations:** a Department of Anesthesiology, West China Hospital, Sichuan University; b Chinese Evidence-based Medicine Center, West China Hospital, Sichuan University, Chengdu, China.

**Keywords:** general anesthesia, geriatric, hip fracture, meta-analysis, regional anesthesia

## Abstract

**Background:**

Previous meta-analyses assessing anesthetic techniques in adult patients undergoing hip fractures surgery are available. However, whether the anesthetic technique is associated with risk of mortality and complications in geriatric patients with hip fractures remains unclear. This study was conducted to assess postoperative outcomes of anesthesia technique in geriatric patients undergoing hip fracture surgery.

**Methods:**

Cochrane Library, PubMed, EMBASE, MEDLINE, CNKI, and CBM were searched from inception up to May 25, 2018. Observational studies and randomized controlled trials (RCTs) that assessed the perioperative outcomes of technique of anesthesia (general or regional [epidural/spinal/neuraxial]) in geriatric patients (≥60 years old) undergoing hip fracture surgery were included. Two investigators independently screened studies for inclusion and performed data extraction. Heterogeneity was assessed by the *I*^2^ and Chi-square tests. The odds ratio (OR) of the dichotomous data, mean difference (MD) of continuous data, and 95% confidence intervals (CI) were calculated to assess the pooled data.

**Results:**

Eleven retrospective and 2 RCTs were included. There was no difference in 30-day mortality (OR = 0.96; 95% CI 0.86–1.08; *P* = .51) between the general and regional anesthesia groups. In-hospital mortality (OR = 1.26; 95% CI 1.17–1.36; *P* < .001), acute respiratory failure (OR = 2.66; 95% CI 2.34–3.02; *P* < .001), length of hospital stay (MD = 0.33; 95% CI 0.24–0.42; *P* < .001), and readmission (OR = 1.09; 95% CI 1.01–1.18; *P* = .03) were significantly reduced in the regional anesthesia group. Pneumonia (OR = 0.99; 95% CI 0.91–1.07; *P* = .79), heart failure (OR = 0.97; 95% CI 0.86–1.09; *P* = .62), acute myocardial infraction (OR = 1.07; 95% CI 0.99–1.16; *P* = .10), acute renal failure (OR = 1.32; 95% CI 0.97–1.79; *P* = .07), cerebrovascular accident (OR = 1.08; 95% CI 0.82–1.42; *P* = .58), postoperative delirium (OR = 1.51; 95% CI 0.16–13.97; *P* = .72), and deep vein thrombosis/pulmonary embolism (OR = 1.42; 95% CI 0.84–2.38; *P* = .19) were similar between the two anesthetic techniques.

**Conclusion:**

General anesthesia is associated with increased risk of in-hospital mortality, acute respiratory failure, longer hospital stays, and higher readmission. There is evidence to suggest that regional anesthesia is associated with improved perioperative outcomes. Large RCTs are needed to explore the most optimal anesthetic techniques for geriatric patients with hip fractures before drawing final conclusions.

**PROSPERO registration number:**

CRD42018093582.

## Abstract 2

Supplemental Digital Content is available in the text

## 1 Introduction

There are approximately 1.66 million hip fractures worldwide each year, and the majority (95%) of hip fracture occurs in patients aged 60 or over,^[1,2]^ it is estimated that there will be 6.26 million hip fractures per year by 2050.^[3]^ Despite efforts to optimize perioperative care of these individuals, evidences show that 30-day mortality in geriatric hip fracture patients approaches 14%, 1-year mortality is up to 17% to 37%,^[4–8]^ and about 20% of patients suffer severe postoperative complications.^[9–11]^ Efforts are needed to improve the quality and outcomes of anesthesia care for these high-risk patients.

Evidence-based clinical practice in geriatric orthopedic anesthesia has been impeded by prior studies showing conflicting results in mortality and postoperative complications depending on anesthesia techniques.^[10,12–14]^ Desai et al^[10]^ performed a retrospective study of geriatric patients with hip fractures, and suggested that regional anesthetic techniques may be preferred with respect to in-hospital mortality and all-cause readmission. In contrast, a randomized controlled trial (RCT) demonstrated that regional anesthesia is associated with an increased risk of 1-year mortality, with no difference in postoperative complications.^[13]^ The prior meta-analysis or studies assessing the effects of anesthesia included all patients with hip fractures regardless of age.^[4,5,8,14–17]^ O’Donnell conducted a meta-analysis, which 32 studies were included, demonstrating no significant difference was observed in 30-day mortality, adverse events for regional compared to general anesthesia.^[5]^ Of which, 14 of 32 studies were focused on adult. Because mortality and morbidity after hip fracture mainly occur in elderly patients, previous studies may have overlooked the important impact of anesthesia exposure on postoperative prognosis in high-risk geriatric hip fracture patients.^[4,5]^ Although some basic standards for orthopedic anesthesia in hip fracture patients have been established in the recently published expert consensus, geriatric patients lack adequate medical attention and the orthogeriatric anesthesia program lacks sufficient clinical evidence.^[18]^ As a result, uncertainty remains regarding the best-practice for geriatric anesthetic management in hip fracture patients.

Based on the aforementioned limitations, this study aimed to evaluate postoperative outcomes of mode of anesthesia [(general anesthesia = general anesthesia only, or combined local anesthesia, or spinal/epidural anesthesia) vs (regional anesthesia = local, spinal, epidural, nerve block)] in geriatric patients underwent hip fracture surgery.

## 2 Methods

The study protocol has not been previously published. The present systematic review and meta-analysis adhered to the preferred reporting items for systematic reviews and meta-analysis guidelines.^[19]^ All analyses were based on previously published studies, thus no ethical approval and patient consent are required. It has been registered in the international prospective register of systematic reviews and the review protocol is available in the supplementary material (Supplementary 1).

### 2.1 Search strategy and criteria

Two investigators (CDX and DL) independently searched the electronic databases Cochrane Library and PubMed. EMBASE, MEDLINE, CNKI, and CBM were searched through OvidSP. In PubMed, the following search strategy was used: ((“hip fractures” [MeSH Terms] OR (“hip” [All Fields] AND “fractures” [All Fields]) OR “hip fractures” [All Fields] OR (“hip” [All Fields] AND “fracture” [All Fields]) OR “hip fracture” [All Fields]) AND (“anesthesia” [MeSH Terms] OR “anesthesia” [All Fields])) AND (“2000/ 01/01” [PDat]: “2018/05/01” [PDat]). The last retrieval was performed on May 25, 2018.

Before carrying out this study, exclusion and inclusion criteria were defined by all authors. This systematic review and meta-analysis focused on the most recent studies evaluating modern anesthetic techniques. Therefore, we included only human studies published between January 1, 2000 and May 25, 2018, assessing perioperative outcomes of different anesthetic techniques (general anesthesia = general anesthesia only, or combined local anesthesia, or spinal/epidural anesthesia) versus (regional anesthesia = local, spinal, epidural, nerve block) in elderly individuals (≥60 years old) with hip fractures. Cohort studies and randomized controlled studies were included, which addressed 30-day mortality and in-hospital mortality, postoperative complications, length of hospital stay, and readmission. All eligible studies were included regardless of sample size. Hand-searching the reference sections of all eligible studies and previously published review articles to identify additional studies.^[4,5,15]^

References were managed using EndNote1 X8 software (Thomson Reuters, New York, NY). Two reviewers (CDX and DL) independently performed an initial screening of titles and abstracts for all retrieved studies. Any dispute was resolved by discussion with a third reviewer (LQ). Two reviewers (CDX and DL) extracted data from articles in a standardized file, and an independent investigator (LQ) validated the extracted data.

### 2.2 Data extraction

A standardized table according to the Population, Intervention, Comparison, Outcome, Study type approach was made^[20]^ and included study characteristics such as author names, study types, participants, anesthesia techniques, primary outcomes, and conclusion. A meta-analysis of 30-day mortality, in-hospital mortality, postoperative complications (pneumonia, respiratory failure, heart failure, acute myocardial infarction, acute renal failure, cerebrovascular accident, postoperative delirium, and deep vein thrombosis/pulmonary embolism [DVT/PE]), length of hospital stay, and readmission was performed. All postoperative complications were reported in eligible studies are presented in Supplementary 2.

### 2.3 Assessment of risk of bias

The methodological quality of non-randomized studies was assessed according to the Newcastle–Ottawa scale (NOS),^[21]^ the 9-item of bias was also divided into high, moderate, or low risk. The risk of bias for each RCT was assessed as suggested by the Cochrane Collaboration Handbook for Systematic Reviews of Interventions.^[22]^ Risk of bias was classified as high, low, and unclear for each of selection bias types, including performance, detection, attrition, reporting, and other biases.

### 2.4 Grading the quality of evidence

The quality of evidence for each finding was rated based on criteria established by the grading of recommendations assessment, development, and evaluation (GRADE) group. Quality of evidence was classified as very low, low, moderate, or high.^[22]^ Any disagreement was settled by discussion among the research team.

### 2.5 Statistical analysis

Review Manager (RevMan for Windows, version 5.3; Cochrane Collaboration, Oxford, UK) and the Stata statistical software version 13.0 (Stata Corp LP, College Station, TX) were used to perform all meta-analyses. The odds ratios (OR) with 95% confidence intervals (CI) for general anesthesia compared with regional anesthesia were calculated for dichotomous variables. The mean differences (MD) and 95% CI were calculated for continuous variables. *P* < .05 was considered statistically significant. *I*^2^ test and Chi-square test were used to assess heterogeneity. Significant heterogeneity was denoted by *I*^2^ > 50% or Chi-square *P* < .1. And leave-out method was used to exclude some trials or subgroup analysis to reduce between-study significant heterogeneity. To validate results, sensitivity analysis was performed to evaluate the stability of these outcomes. However, due to the relatively small number of randomized studies, to avoid the unreliability of the random-effects model, we adopted a fixed-effects model for sensitivity analysis to evaluate the stability of these results. Both the Begg and Egger regression tests were performed using the meta-bias command in STATA, and funnel plots were constructed to detect publication bias.^[20]^

## 3 Results

### 3.1 Study selection

**Figure 1:**
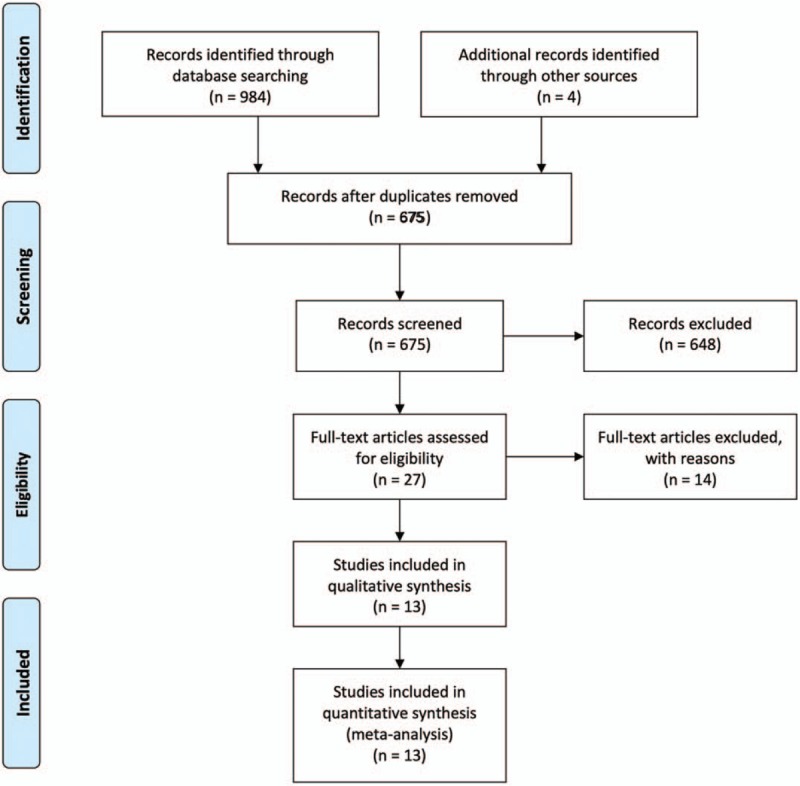
PRISMA-flow diagram for the literature search and exclusion criteria. PRISMA = preferred reporting items for systematic reviews and meta-analysis.

Initially, 988 studies were identified. After removal of duplicates, 675 titles and abstracts were screened. Excluding 648 articles according to title and abstract review, 27 reports were retrieved for full-text review. Finally, 13 studies, including eleven retrospective observational studies, 2 RCTs, evaluating 196,646 patients were included^[9,10,16,23–32]^; A flow diagram depicting the selection process of eligible studies is shown in Figure 1.

### 3.2 Study characteristics/participants

Table 1 displayed the detailed characteristics of the studies. Of the 13 studies that entered the analysis, 11 were retrospective observational studies involving 196,571 patients and the other 2 RCTs had only 75 cases.^[9,10,16,23–32]^ And of the total 196,646 patients, 104,364 received general anesthesia and 91,237 received local anesthesia, respectively. The sample size of the included studies ranged from 30 to 104,088. Three studies examined the outcomes with general and regional anesthesia,^[10,25,28]^ 6 studies focused on outcomes of spinal compared with general anesthesia,^[9,24,26,29,30,32]^ 3 studies evaluated the results of spinal and/or epidural anesthesia and general anesthesia^[16,23,27]^ and one trial did not provide the definition of regional anesthesia.^[31]^

**Table 1:**
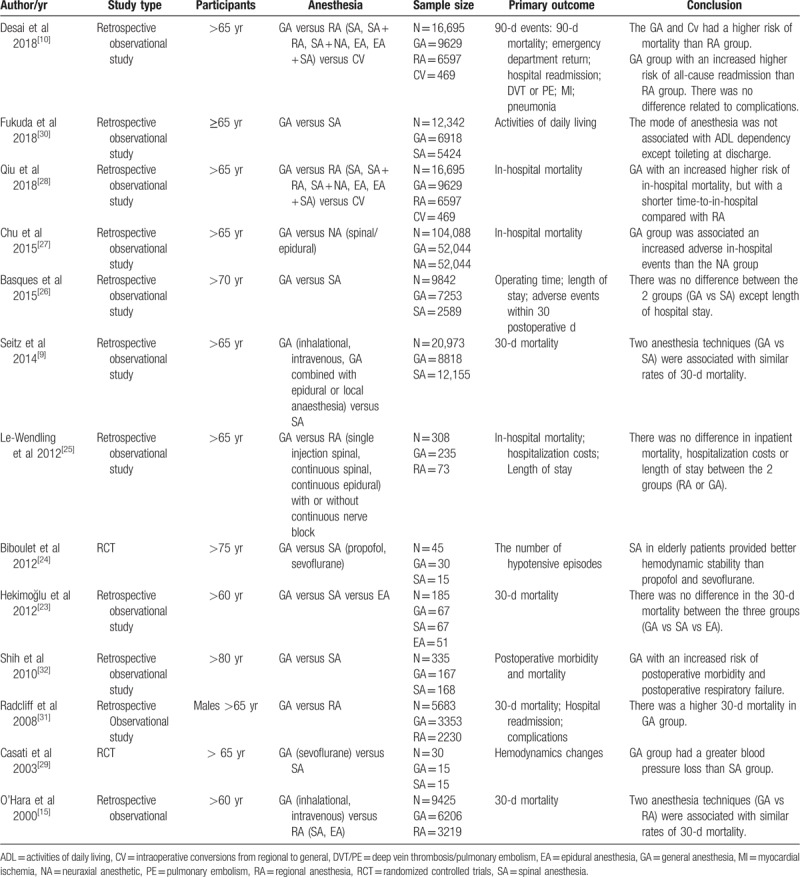
Demographic characteristics of the included studies.

### 3.3 Risk of bias and quality of evidence

**Figure 2:**
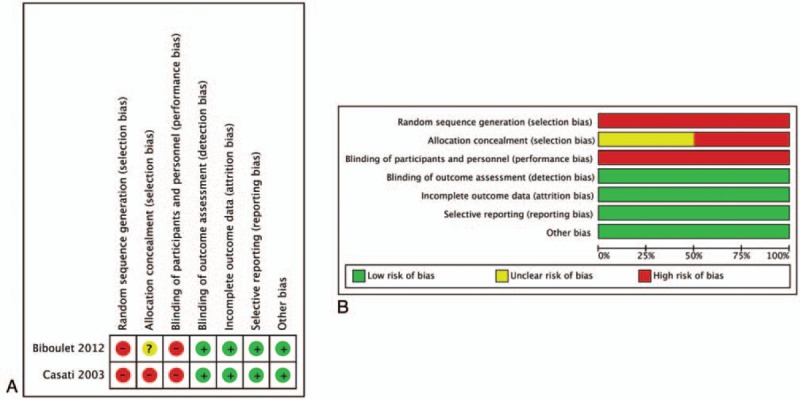
(A) Risk of bias summary: review authors’ judgements about each risk of bias item for each included study. (B) Risk of bias graph: review authors’ judgements about each risk of bias item presented as percentages across all included studies.

In the observational study, NOS scores ≥6 were considered high quality and all included studies met high-quality criteria (Table 2). Regarding the 2 RCTs, the risk of selection bias and performance bias was high because of the lack of information on whether or how to use blinding during intervention and outcome assessment. In this meta-analysis, attrition, and reporting bias were low (Fig. 2). The GRADE quality of evidence was presented in Appendix 1.

**Table 2:**
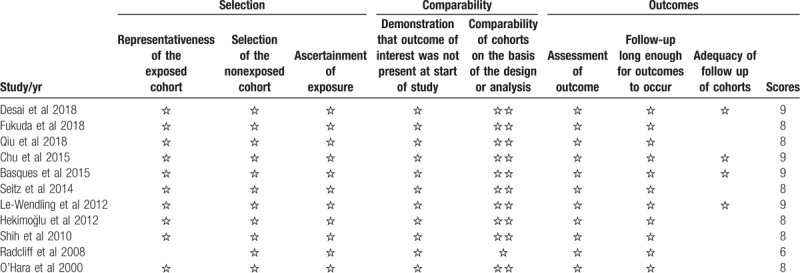
Summary of data comparing general and regional anesthesia (postoperative complications, length of hospital stay, and readmission rate).

### 3.4 30-day mortality/in-hospital mortality

**Figure 3:**
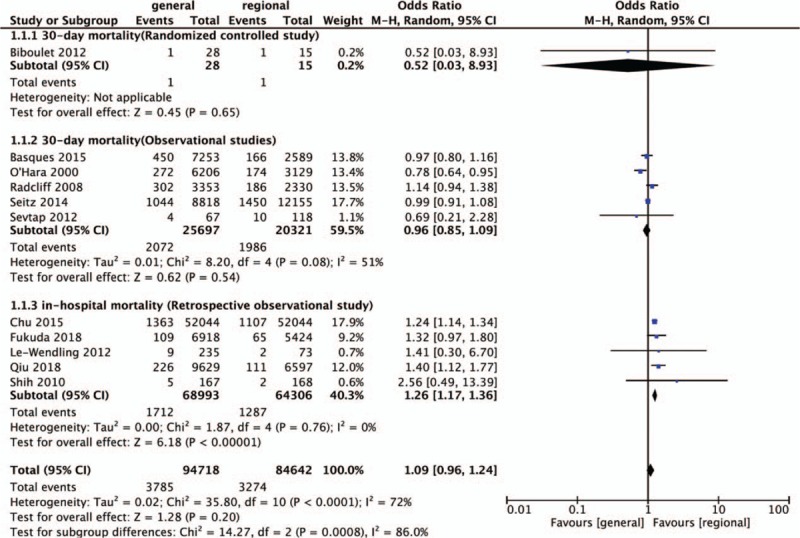
Meta-analysis of the 30-d mortality in-hospital mortality for the general anesthesia group versus the regional anesthesia group.

Eleven studies evaluated the effect of general versus regional anesthesia on all-cause 30-day and in-hospital mortality after hip surgery in geriatric patients, including 10 retrospective observational studies^[9,10,16,23,25–27,30–32]^ and 1 RCT.^[24]^ The results are presented in Figure 3.

**Figure 4:**
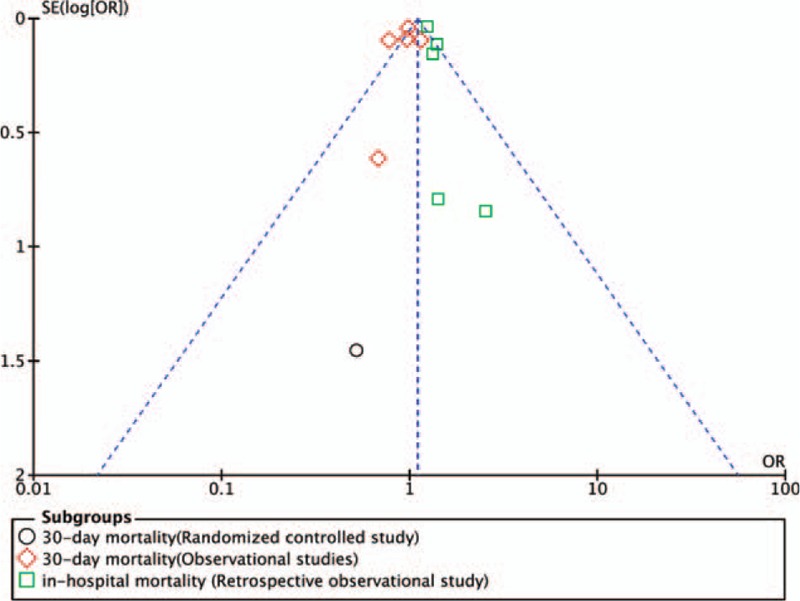
The funnel plot of 30-d/in-hospital mortality suggested that there was no publication bias, which was also statistically supported by the Egger test (*P* = .88) and Begg test (*P* = .70).

The meta-analysis including 6 studies (1 RCT^[24]^ and 5 retrospective observational studies^[9,16,23,26,31]^) revealed no significant difference in the 30-day mortality (OR = 0.96; 95% CI 0.86–1.08; *I*^*2*^ = 40%; *P* = .51, n = 46,061). The GRADE quality of evidence was moderate (Appendix 1) and the publication bias as assessed by visual inspection of the funnel plot (Fig. 4). A further subgroup analysis between the 5 observational studies (OR = 0.96; 95% CI 0.85–1.09; *P* = .54, n = 46,018) and 1 RCT (OR = 0.52; 95% CI 0.03–8.93; *P* = .65, n = 43) also revealed no significant difference for 30-day mortality.

Five retrospective observational studies showed a significantly higher in-hospital mortality in the general anesthesia group (OR = 1.26; 95% CI 1.17–1.36; *I*^*2*^ = 0%; *P* < .001, n = 133,299).^[25,27,28,30,32]^ The GRADE quality of evidence was high (Appendix 1), and the funnel plot was shown in Figure 4. The Egger test (*P* = .88), and the Begg test (*P* = .70) demonstrated no evidence of small study publication bias.

### 3.5 Complications

All included studies reported various perioperative complications, which were categorized and illustrated in Supplementary 2. Based on comparable information extracted, quantitative analysis was performed, and results are shown below (Table 3).

**Table 3:**
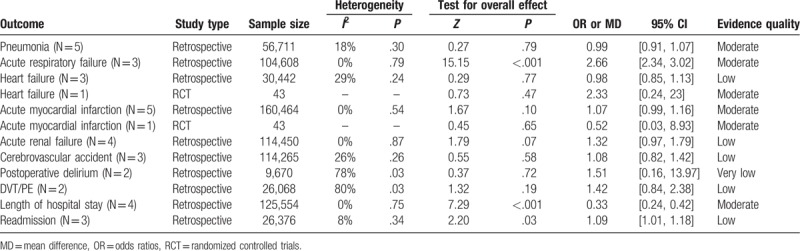
Summary of data comparing general and regional anesthesia (postoperative complications, length of hospital stay, and readmission rate).

#### 3.5.1 Pneumonia

Five retrospective observational studies reported pneumonia incidence.^[9,10,16,26,32]^ The current meta-analysis indicated similar pneumonia rates in the general and regional anesthesia groups (OR = 0.99; 95% CI 0.91–1.07; *I*^2^ = 18%; *P* = .79, n = 56,711; Table 3).

#### 3.5.2 Acute respiratory failure

Three retrospective observational studies evaluated the incidence of acute respiratory failure after general and regional anesthesia in geriatric patients with hip fractures.^[23,27,32]^ The meta-analysis reported a significant higher rate of acute respiratory failure in the general anesthesia group (OR = 2.66; 95% CI 2.34–3.02; *I*^2^ = 0%; *P* < .001, n = 104,608; Table 3).

#### 3.5.3 Heart failure

Heart failure incidence was assessed by 3 retrospective observational studies and 1 RCT,^[9,16,23,24]^ involving 15,119 general and 15,366 regional anesthesia patients; the results showed that there was no significant difference in heart failure incidence (OR = 0.97; 95% CI 0.86–1.09; *I*^2^ = 12%; *P* = .62, n = 30,485; Table 3). A subgroup analysis between the 3 retrospective observational studies (OR = 0.98; 95% CI 0.85–1.13; *P* = .77) and 1 RCT (OR = 2.33; 95% CI 0.24–23.00; *P* = .47) also indicated no significant difference in heart failure incidence concerning the general and regional anesthesia.

#### 3.5.4 Acute myocardial infarction

Five retrospective observational studies and one RCT assessed acute myocardial infarction,^[9,10,16,24,26,27]^ and found no significant difference in myocardial infarction rate in patient receiving general and regional anesthesia (OR = 1.07; 95% CI 0.99–1.16; *I*^*2*^ = 0%; *P* = .10, n = 160,507; Table 3). And the subgroup analysis between the retrospective observational studies and the RCT also indicated no significant difference in acute myocardial infarction rate between the 2 types of anesthesia (OR = 0.52; 95% CI 0.03–8.93; *P* = .65) versus (OR = 1.07; 95% CI 0.99–1.16; *P* = .10).

#### 3.5.5 Acute renal failure

The incidence of acute renal failure was examined by 4 retrospective observational studies,^[23,26,27,32]^ in a total of 114,450 patients. Pooled analysis indicated no significant difference in acute renal failure (OR = 1.32; 95% CI 0.97–1.79; *I*^*2*^ = 0%; *P* = .07; n = 114,450; Table 3).

#### 3.5.6 Cerebrovascular accident

Three retrospective observational studies assessed the total rate of cerebrovascular accident after hip fracture surgery in geriatric patients,^[26,27,32]^ including 59,464 general and 54,801 regional anesthesia cases. There was no significant difference in cerebrovascular accident between general and regional anesthesia (OR = 1.08; 95% CI 0.82–1.42; *I*^2^ = 26%; *P* = .58, n = 114,265; Table 3).

#### 3.5.7 Postoperative delirium

Only 2 retrospective observational studies estimated postoperative delirium.^[16,32]^ A total of 9670 geriatric patients were analyzed altogether, and no significant difference in postoperative delirium was observed, with statistically significant heterogeneity (OR = 1.51; 95% CI 0.16–13.97; *I*^*2*^ = 78%; *P* = .72; n = 9670; Table 3).

#### 3.5.8 DVT/PE

As for DVT/PE, only 2 large retrospective observational studies were involved.^[10,26]^ Pooled analysis showed no significant difference, with statistically significant heterogeneity (OR = 1.42; 95% CI 0.84–2.38; *I*^2^ = 80%; *P* = .19, n = 26,068; Table 3).

#### 3.5.9 Length of hospital stay

The length of hospital stay was assessed in four retrospective observational studies.^[9,23,25,27]^ Pooled analysis revealed that general anesthesia was associated longer hospital stay (MD = 0.33; 95% CI 0.24–0.42; *I*^2^ = 0%; *P* < .001; n = 125,554; Table 3).

#### 3.5.10 Readmission

Three retrospective observational studies evaluated readmission.^[10,25,26]^ The meta-analysis reported a significant higher rate of readmission in the general anesthesia group (OR = 1.09; 95% CI 1.01–1.18; *I*^*2*^ = 8%; *P* = 0.03; n = 26,376; Table 3).

### 3.6 Sensitivity analysis

Five observational studies^[9,16,23,26,31]^ revealed no significant difference in the 30-day mortality (OR = 0.98; 95% CI 0.91–1.04; *P* = .51). For in-hospital mortality, 5 observational studies^[10,25,27,30,32]^ indicated general anesthesia showing a higher rate of in-hospital mortality compared to the regional anesthesia (OR = 1.25; 95% CI 1.15–1.35; *P* < .001). Five observational studies^[9,10,16,26,32]^ showed no significant difference for prevalence of pneumonia (OR = 0.99; 95% CI 0.93–1.06; *P* = .82). Three observational studies^[23,27,32]^ showed that general anesthesia was associated with an increased risk of acute respiratory failure compared with regional anesthesia (OR = 3.66; 95% CI 2.35–3.02; *P* < .001). Three observational studies and one RCT^[9,16,23,24]^ evaluated the incidence of heart failure, demonstrating that no significant difference was observed between the 2 anesthesia types (OR = 0.95; 95% CI 0.87–1.04; *P* = .26). Five observational studies^[9,10,16,26,27]^ revealed that no significant difference in the incidence of acute myocardial infarction (OR = 1.07; 95% CI 0.99–1.16; *P* = .09). About acute renal failure, four observational studies^[23,26,27,32]^ shown no significant difference between general and regional anesthesia (OR = 1.32; 95% CI 0.98–1.79; *P* = .07). Three observational studies^[26,27,32]^ demonstrated that general anesthesia was associated higher incidence of cerebrovascular accident (OR = 1.16; 95% CI 1.05–1.28; *P* = .003). Two observational studies^[16,32]^ showed general anesthesia was associated a reduced risk of postoperative delirium (OR = 0.61; 95% CI 0.56–0.67; *P* < .001). Two observational studies^[10,26]^ showed that a statistically significant difference for DVE/PE (OR = 1.26; 95% CI 1.05–1.51; *P* = .01). Four observational studies^[9,23,25,27]^ indicated that general anesthesia was related to a prolonged length of hospital stay (MD = 0.33; 95% CI 0.24–0.42; *P* < .001). Based on 3 observational studies,^[10,25,26]^ we found general anesthesia had a higher readmission compared with regional anesthesia (OR = 1.09; 95% CI 1.02–1.17; *P* = .01).

## 4 Discussion

In the present systematic review and meta-analysis, we included 13 studies (11 retrospective studies and 2 RCTs), with 196,646 patients, in which 104,364 patients received general anesthesia and 91,244 received regional anesthesia. The results showed that for geriatric patients undergoing hip fracture surgery, general anesthesia was associated with an increased risk of in-hospital mortality compared with regional anesthesia, as well as the risk of acute respiratory failure, length of hospital stay, and readmission. However, the significant difference was not achieved in this study for postoperative pneumonia, heart failure, acute myocardial infarction, acute renal failure, cerebrovascular accident, delirium, and DVT/PE.

In 2016, Guay and colleagues performed a systematic review,^[33]^ which included 31 RCTs published between 2003 and 2014 to assess the role of different anesthesia techniques in hip fracture surgery. However, only 11 RCTs provided 2152 patients to examine 30-day mortality, obviously, the sample size was insufficient to determine a difference between general and regional anesthesia. And the authors noted that the anesthesia techniques used in some of the studies they included in the meta-analysis may not reflect current clinical practice, preventing them from finding reliable results. Later, Julia et al^[4]^ and O’Donnell et al^[5]^ conducted 2 systematic reviews in 2016 and 2017, respectively, to assess the type of anesthesia in adult patients undergoing hip fracture surgery. Both reviews included RCTs and observational studies, increasing the number of patients to 413,999 and 202,000, respectively. Our study also include both prospective RCTs and retrospective observational studies, in contrast, we focused on the elderly population (≥60 years) and included three new retrospective studies published in 2018.^[10,28,30]^

Five retrospective observational studies and 1 RCT evaluated the 30-day mortality. And none of the studies showed significant difference in 30-day mortality between the 2 groups.^[9,16,23,24,26,31]^ It is worth noting that the RCT performed by Biboulet and colleagues included only 45 patients,^[24]^ while the other 5 observational studies included a total of 46,018 patients,^[9,16,23,26,31]^ so the latter got the greatest weight in the analysis. Therefore, the possibility of bias associated with observational study design should be considered as it may have a significant impact on the results of this meta-analysis. Large prospective RCTs of general and regional anesthesia are urgently needed to provide a basis for clinical guidelines.

Meanwhile, 5 other retrospective observational studies involved 133,299 patients to evaluate the in-hospital mortality.^[25,27,28,30,32]^ And the meta-analysis revealed that general anesthesia is associated with an increased risk of in-hospital mortality. According to our calculations, powering a randomized study to determine a difference in in-hospital mortality at the minimum acceptable power criteria of 80% would require only 5082 patients per group. However, our data of the in-hospital mortality is also derived from observational studies, so the study can only identify associations, but cannot conclude the causal relationships. In addition, this meta-analysis reveals that the results between 30-day mortality and in-hospital mortality are inconsistent, suggesting that anesthesia techniques have a greater impact on short-term outcomes than long-term outcomes.

The present meta-analysis examined postoperative complications, and only found a higher incidence of acute respiratory failure in geriatric patients receiving general anesthesia group. Three studies assessed the acute respiratory failure,^[23,27,32]^ and the conclusion remained unchanged after sensitivity analysis. However, Chu and colleagues’ study included 104,088 patients, which accounted for 99.5% of the weight of the meta-analysis.^[27]^ The other 2 studies are quite small.^[23,32]^ Given this limitation, our results need to be interpreted with caution. The meta-analysis showed no significant differences in prevalence of pneumonia, heart failure, acute myocardial infarction, acute renal failure, cerebrovascular accident, postoperative delirium, and DVT/PE. But the sensitivity analysis in cerebrovascular accident, postoperative delirium and DVE/PE revealed that the results from the meta-analysis were generally unstable. Large heterogeneity in the definition of postoperative complications may be mainly responsible for the unstable results. For example, 1 study^[10]^ defined cerebrovascular accident and DVT/PE as quality indicators according to the Agency for Healthcare Research and Quality, while another^[26]^ was accord to the American College of Surgeons National Surgical Quality Improvement Program to define DVT/PE. Similarly, 1 study^[16]^ defined the delirium as sleepiness or a change in mental status, and another study^[32]^ did not provide the definition of delirium. Unclear definition may lead to a wide prevalence range for this outcome, ranging from 2.1%^[5]^ to 28.7%.^[15]^ Moreover, most of the pooled data on postoperative complications were derived from retrospective observational studies, and due to the lack of sufficient powered RCT and the risk of confounding in the observational studies, no authoritative conclusion could be drawn.

Previous systematic review and meta-analysis which included only RCTs^[33]^ concluded that no significant difference was found in hospital stays between general and regional anesthesia groups in adult patients. In the present meta-analysis, we included both observational studies and RCTs for geriatric patients and found that there was a statistical significance favoring regional anesthesia for length of hospital stay. Among the included studies, Basques et al^[26]^ found that general anesthesia is associated with shorter length of hospital stay. Conversely, Shih et al^[32]^ and Chu et al^[27]^ revealed general anesthesia was associated with longer hospital stay compared with regional anesthesia and these 2 studies got the most weight in the analysis thus affecting the final results. Another limitation was that only Basques's study provided the definitions of the length of hospital stay as the days from operation to discharge,^[26]^ but provided no information regarding the total length of stay and other studies did not disclose their definitions of length of stay.^[9,23,25,27]^ Additionally, we found that general anesthesia only increased the average length of hospital stay by about 0.33 days, suggesting that clinical significance is small.

From current data, we found that general anesthesia is associated with a higher risk of readmissions compared with regional anesthesia. Previous studies by Le-Wendling et al^[25]^ and Basques et al^[26]^ found no difference in readmissions rates; however, these studies recorded readmissions within the first 30 days but Desai et al^[10]^ estimated the readmission within 90-day, demonstrating that general anesthesia was associated an increased risk of readmission. Besides, the latter got the greatest weight in the analysis. Moreover, in Le-Wendling et al's studies,^[25]^ 30-day readmission was determined by whether patients were readmitted to a Veterans Health Administration hospital or the same hospital, respectively, rather than any hospital, which it could contribute to a measurement bias. Given aforementioned, whether the finding favors regional anesthesia with less readmission is uncertain.

Anesthesia is an indispensable part of the multidisciplinary care of hip fracture patients. Healthcare providers need to select the optimal anesthetic technique for hip fracture surgery after comprehensively evaluating the full clinical picture rather than relying on personal preference. Regional anesthesia is often the recommended anesthetic technique in hip fracture surgery,^[34,35]^ and increasing evidence shows regional anesthesia may be preferable.^[6]^ However, a recent study found that increasing the frequency of neuraxial anesthesia use is not associated with reduced complications and the length of hospital stay.^[36]^ While still debated, expert consensus or healthcare policy have inclined to recommend applying regional anesthesia in hip fracture recipients.^[18,37]^ But the fact remains that except to an increasing literature suggesting superiority, no evidence of inferiority of general versus regional anesthesia exists in fact.^[15]^ To date, no conclusions can be drawn on how perioperative outcomes are affected by the utilization of regional anesthesia. Certainly, the focus of future studies must shift from reporting ambiguous defined outcomes to performing research that includes well-defined interventions and patient-important perioperative outcome. Improve hip fracture outcome in the elderly patient: a multicenter RCT to test the efficacy of spinal versus general anesthesia is good examples of ongoing research where some of these principles have been considered.^[38]^

### 4.1 Limitations

Several limitations of this meta-analysis should be mentioned. First, current evidence lacks RCTs of high quality, the most of enrolled studies our study was retrospective and inherently limited by the quality of the available study. Second, Supplementary 2, indicates that perioperative complications were prevalent in geriatric patients with hip fracture surgery, but the postoperative complications lack uniform detailed definitions and validated diagnostic criteria. As a result, most studies were not included in meta-analysis. Besides, the types of complications reported in various studies were inconsistent, and the possibility of negative outcomes remaining unpublished could not be ruled out.

## 5 Conclusion

In this meta-analysis, we could not observe any difference in the 30-day mortality rate between regional and general anesthesia. We did, however, find that patients receiving regional anesthesia have shorter in-hospital mortality, acute respiratory failure, readmission, and hospital stay than patients undergoing general anesthesia. These results support that regional anesthesia is associated with improved perioperative outcomes.

## Acknowledgments

We would especially express thanks to editors at MedSci for language editing. We would like to thank all the authors in particular.

## Author contributions

**Conceptualization:** Dong Xu Chen, Lei Yang, Qian Li.

**Data curation:** Dong Xu Chen, Lin Ding.

**Formal analysis:** Dong Xu Chen.

**Funding acquisition:** Qian Li.

**Investigation:** Dong Xu Chen.

**Methodology:** Dong Xu Chen, Lei Yang, Lin Ding, Ya Na Qi.

**Project administration:** Dong Xu Chen, Lin Ding, Shi Yue Li.

**Resources:** Dong Xu Chen, Lin Ding, Shi Yue Li.

**Software:** Dong Xu Chen.

**Supervision:** Ya Na Qi, Qian Li.

**Validation:** Lei Yang, Ya Na Qi, Qian Li.

**Writing – original draft:** Dong Xu Chen.

**Writing – review and editing:** Dong Xu Chen, Lei Yang, Qian Li.

## Supplementary Material

Supplemental Digital Content

## Supplementary Material

Supplemental Digital Content

## Supplementary Material

Supplemental Digital Content
