# Supplementary material for: Perioperative outcomes in geriatric patients undergoing hip fracture surgery with different anesthesia techniques: A systematic review and meta-analysis
Source: Medicine (Baltimore). 2019 Dec 10;98(49):e18220. doi: 10.1097/MD.0000000000018220 (PMC6919429; doi:10.1097/MD.0000000000018220)
Supplement: Supplemental Digital Content [file medi-98-e18220-s003.pdf]

Author(s):
Date: 2018-06-20
Question: Should Regional vs. General anesthesia vs Regional vs. General anesthesia be used in Hip fractures geriatric patients (â‰¥ 60 years old)?
Settings: Different anesthesia technique
Bibliography: Cochrane Database of Systematic Reviews

Quality assessment							No of patients		Effect		Quality	Importance
**No of studies**	**Design**	**Risk of bias**	**Inconsistency**	**Indirectness**	**Imprecision**	**Other considerations**	**Regional vs. General anesthesia**	**Regional vs. General anesthesia**	**Relative (95% CI)**	**Absolute**		
**30-day mortality**												
5	observational studies	no serious risk of bias	serious[1]	no serious indirectness	no serious imprecision	strong association[2] increased effect for RR ~1[3]	2073/25725 (8.1%)	1987/20336 (9.8%)	OR 0.96 (0.86 to 1.08)	4 fewer per 1000 (from 13 fewer to 7 more)	⊕⊕⊕O MODERATE	CRITICAL
								7.3%		3 fewer per 1000 (from 10 fewer to 5 more)		
**30-day mortality**												
1	randomised trials	serious[4]	no serious inconsistency	no serious indirectness	no serious imprecision	none	1/28 (3.6%)	1/15 (6.7%)	OR 0.52 (0.03 to 8.93)	31 fewer per 1000 (from 65 fewer to 323 more)	⊕⊕⊕O MODERATE	IMPORTANT
								0%		-		
**in-hospital mortality**												
5	observational studies	no serious risk of bias	no serious inconsistency	no serious indirectness	no serious imprecision	strong association[2] increased effect for RR ~1[3]	2708/68993 (3.9%)	1916/64306 (3%)	OR 1.21 (1.14 to 1.28)	6 more per 1000 (from 4 more to 8 more)	⊕⊕⊕⊕ HIGH	CRITICAL
								2.1%		4 more per 1000 (from 3 more to 6 more)		

[1] The sample content varies greatly
[2] this is a big sample
[3] use the propensity score matching to reduce select bias
[4] It's not clear what the random allocation is

Author(s):
Date: 2018-06-20
Question: Should Regional vs. General anesthesia vs Regional vs. General anesthesia be used in Hip fractures geriatric patients (â‰¥ 60 years old)?
Settings: Different anesthesia technique
Bibliography: Cochrane Database of Systematic Reviews

Quality assessment							No of patients		Effect		Quality	Importance
**No of studies**	**Design**	**Risk of bias**	**Inconsistency**	**Indirectness**	**Imprecision**	**Other considerations**	**Regional vs. General anesthesia**	**Regional vs. General anesthesia**	**Relative (95% CI)**	**Absolute**		
**pneumonia**												
5	observational studies	no serious risk of bias	no serious inconsistency	no serious indirectness	no serious imprecision	strong association[1]	2107/32073 (6.6%)	1785/24638 (7.2%)	OR 0.99 (0.91 to 1.07)	1 fewer per 1000 (from 6 fewer to 5 more)	⊕⊕⊕O MODERATE	IMPORTANT
								4.2%		0 fewer per 1000 (from 4 fewer to 3 more)		
**acute respiratory failure**												
3	observational studies	no serious risk of bias	no serious inconsistency	no serious indirectness	no serious imprecision	increased effect for RR ~1[2]	877/52278 (1.7%)	336/52330 (0.6%)	OR 2.66 (2.34 to 3.02)	10 more per 1000 (from 8 more to 13 more)	⊕⊕⊕O MODERATE	IMPORTANT
								0.6%		10 more per 1000 (from 8		

										more to 12 more)		
**acute renal failure**												
4	observational studies	no serious risk of bias	no serious inconsistency	no serious indirectness	no serious imprecision	none	111/59531 (0.2%)	70/54919 (0.1%)	OR 1.32 (0.98 to 1.79)	0 more per 1000 (from 0 fewer to 1 more)	⊕⊕OO LOW	IMPORTANT
								0.8%		3 more per 1000 (from 0 fewer to 6 more)		
**heart failure**												
1	randomised trials	serious[3]	no serious inconsistency	no serious indirectness	no serious imprecision	none[2]	4/28 (14.3%)	1/15 (6.7%)	OR 2.33 (0.24 to 23)	76 more per 1000 (from 50 fewer to 555 more)	⊕⊕⊕O MODERATE	IMPORTANT
								0.6%		8 more per 1000 (from 5 fewer to 116 more)		
**heart failure**												
3	observational studies	no serious risk of bias[3]	no serious inconsistency	no serious indirectness	no serious imprecision	none[2]	1091/15091 (7.2%)	1317/15351 (8.6%)	OR 0.98 (0.85 to 1.13)	2 fewer per 1000 (from 12 fewer to 10 more)	⊕⊕OO LOW	IMPORTANT
								0.6%		0 fewer per 1000 (from 1 fewer to 1 more)		
**DVE/PE**												
2	observational studies	no serious risk of bias	no serious inconsistency	no serious indirectness	no serious imprecision	none	388/16882 (2.3%)	179/9186 (1.9%)	OR 1.42 (0.84 to 2.38)	8 more per 1000 (from 3 fewer to 26 more)	⊕⊕OO LOW	IMPORTANT
								1.7%		7 more per 1000 (from 3 fewer to 23 more)		
**postoperative delirium**												
2	observational studies	no serious risk of bias	serious[4]	no serious indirectness	no serious imprecision	none	1571/6373 (24.7%)	1115/3297 (33.8%)	OR 1.51 (0.16 to 13.97)	97 more per 1000 (from 263 fewer to 539 more)	⊕OOO VERY LOW	NOT IMPORTANT
								18.1%		69 more per 1000 (from 147 fewer to 574 more)		
**cerebrovascular accident**												
3	observational studies	no serious risk of bias	no serious inconsistency	no serious indirectness	no serious imprecision	none	892/59464 (1.5%)	740/54801 (1.4%)	OR 1.08 (0.82 to 1.42)	1 more per 1000 (from 2 fewer to 6 more)	⊕⊕OO LOW	IMPORTANT
								0.9%		1 more per 1000 (from 2 fewer to 4 more)		
**acute myocardial infarction**												
5	observational studies	no serious	no serious inconsistency	no serious indirectness	no serious imprecision	increased effect for RR ~1[2]	1314/83978 (1.6%)	1323/76529 (1.7%)	OR 1.07 (0.99 to 1.16)	1 more per 1000 (from 0	⊕⊕⊕O MODERATE	IMPORTANT

		risk of bias								fewer to 3 more)		
								1.9%		1 more per 1000 (from 0 fewer to 3 more)		
**acute myocardial infarction**												
1	randomised trials	serious[3]	no serious inconsistency	no serious indirectness	no serious imprecision	none[2]	1/28 (3.6%)	1/15 (6.7%)	OR 0.52 (0.03 to 8.93)	31 fewer per 1000 (from 65 fewer to 323 more)	⊕⊕⊕O MODERATE	IMPORTANT
								0.6%		3 fewer per 1000 (from 6 fewer to 45 more)		

[1] this is a big sample

[2] use the propensity score matching to reduce select bias

[3] It's not clear what the random allocation is

[4] There's a big sample difference

Author(s):
Date: 2018-06-20
Question: Should Regional vs. General anesthesia vs Regional vs. General anesthesia be used in Hip fractures geriatric patients (â‰¥ 60 years old)?
Settings: Different anesthesia technique
Bibliography: Cochrane Database of Systematic Reviews

Quality assessment							No of patients		Effect		Quality	Importance
No of studies	Design	Risk of bias	Inconsistency	Indirectness	Imprecision	Other considerations	Regional vs. General anesthesia	Regional vs. General anesthesia	Relative (95% CI)	Absolute		
**length of hospital stay (Better indicated by lower values)**												
4	observational studies	no serious risk of bias	no serious inconsistency	no serious indirectness	no serious imprecision	increased effect for RR ~1[1]	61164	64390	-	MD 0.6 lower (2.64 lower to 1.45 higher)	⊕⊕⊕O MODERATE	CRITICAL
**readmission**												
3	observational studies	no serious risk of bias	no serious inconsistency	no serious indirectness	serious	increased effect for RR ~1[2]	2842/17117 (16.6%)	1575/9259 (17%)	OR 1.09 (1.01 to 1.18)	13 more per 1000 (from 1 more to 25 more)	⊕⊕OO LOW	IMPORTANT
								20.1%		14 more per 1000 (from 2 more to 28 more)		

[1] use the propensity score matching to reduce select bias

[2] use the prepensity score matching to reduce select bias
